# Effect of aging on pulmonary cellular responses during mechanical ventilation

**DOI:** 10.1172/jci.insight.185834

**Published:** 2025-02-13

**Authors:** Aminmohamed Manji, Lefeng Wang, Cynthia M. Pape, Lynda A. McCaig, Alexandra Troitskaya, Onon Batnyam, Leah J.J. McDonald, C. Thomas Appleton, Ruud A.W. Veldhuizen, Sean E. Gill

**Affiliations:** 1 Centre for Critical Illness Research, London Health Sciences Centre Research Institute, London, Ontario, Canada.; 2 Department of Physiology and Pharmacology,; 3 Department of Medicine, and; 4 Department of Pathology and Laboratory Medicine, Schulich School of Medicine and Dentistry, Western University, London, Ontario, Canada.

**Keywords:** Aging, Pulmonology, Vascular biology, Endothelial cells, Macrophages, Pulmonary surfactants

## Abstract

Acute respiratory distress syndrome (ARDS) results in substantial morbidity and mortality, especially in elderly people. Mechanical ventilation, a common supportive treatment for ARDS, is necessary for maintaining gas exchange but can also propagate injury. We hypothesized that aging leads to alterations in surfactant function, inflammatory signaling, and microvascular permeability within the lung during mechanical ventilation. Young and aged male mice were mechanically ventilated, and surfactant function, inflammation, and vascular permeability were assessed. Additionally, single-cell RNA-Seq was used to delineate cell-specific transcriptional changes. The results showed that, in aged mice, surfactant dysfunction and vascular permeability were significantly augmented, while inflammation was less pronounced. Differential gene expression and pathway analyses revealed that alveolar macrophages in aged mice showed a blunted inflammatory response, while aged endothelial cells exhibited altered cell-cell junction formation. In vitro functional analysis revealed that aged endothelial cells had an impaired ability to form a barrier. These results highlight the complex interplay between aging and mechanical ventilation, including an age-related predisposition to endothelial barrier dysfunction, due to altered cell-cell junction formation, and decreased inflammation, potentially due to immune exhaustion. It is concluded that age-related vascular changes may underlie the increased susceptibility to injury during mechanical ventilation in elderly patients.

## Abstract 2

Aging leads to increased susceptibility to blood vessel damage and leak within the lungs during mechanical ventilation occuring independently of the inflammatory response.

## Introduction

Acute respiratory distress syndrome (ARDS) is a serious disease with substantially elevated morbidity and mortality in the elderly population (1, 2). Treatment of patients with ARDS is generally supportive, including mechanical ventilation to sustain respiratory function (3). However, advanced age has been suggested to be associated with worse outcomes following mechanical ventilation (4–7). This is supported by animal studies showing that mechanical ventilation can also cause or propagate lung injury, which is termed ventilator-induced lung injury (VILI) (8–11). While insight has been obtained from animal models of VILI, very few studies have used appropriately aged preclinical models to identify and assess cellular and molecular mechanisms (12–17). Given that age is a significant predictor of time spent on a mechanical ventilator and that advanced age is a strong negative predictor of survival, more studies using age-appropriate animal models are critical (4, 5, 7, 18–20).

The mechanisms associated with respiratory dysfunction during VILI in young animals have been well documented and include several pathophysiological processes. The ventilation-induced overdistension of both alveolar epithelial cells and resident alveolar macrophages leads to cellular damage and the initiation of inflammation (21–26). This initial inflammatory response further augments cytokine production, leading to ongoing recruitment of inflammatory cells as well as the propagation of overall lung injury (25). Additionally, mechanical ventilation affects pulmonary surfactant function. Surfactant, which is produced by the alveolar type II cells, reduces surface tension within the lungs while also modulating the pulmonary inflammatory response (27–30). Damage to the alveolar epithelium, due to augmented inflammation and alveolar overdistension, is thought to result in impaired surfactant function (31–36). Cyclic alveolar stretch during VILI also promotes the conversion of functional large aggregate forms of surfactant into nonfunctional small aggregate forms (24, 37, 38).

Another critical process is damage to the microvascular barrier, where VILI subjects the lung vascular endothelium to severe mechanical forces and persistent inflammation, leading to the destruction of the endothelial barrier (39–42). Specifically, VILI disrupts key cell-cell junctional proteins such as claudin-5 in endothelial cells, leading to increased microvascular permeability (43). This increased permeability and the subsequent formation of proteinaceous edema fluid within the alveolar space is also known to contribute to the loss of surfactant function (33, 44–46).

Aging is known to independently affect many of these pathophysiological processes. For example, aging is associated with heightened baseline inflammation and alveolar macrophage dysfunction (47–52). Aging also contributes to vascular endothelial (VE) cell dysfunction and augmented vascular permeability in the heart, kidney, and gut vasculature (53–59). Limited studies, however, have examined the effect of aging within the pulmonary vasculature. Thus, while the effects of VILI and aging on inflammation, alveolar epithelial cell dysfunction, and microvascular endothelial cell barrier dysfunction have been previously investigated independently, a better understanding of the interactions between aging and mechanical ventilation during VILI — and how they influence these responses — is needed. We hypothesized that aging leads to alterations in surfactant function, inflammatory signaling, and microvascular permeability within the lung during mechanical ventilation. To address this hypothesis, we mechanically ventilated young and aged male mice and used single-cell RNA-Seq (scRNA-Seq) in combination with various biochemical methods to assess these pathophysiological processes.

## Results

### Physiological responses and lung histology.

**Figure 1:**
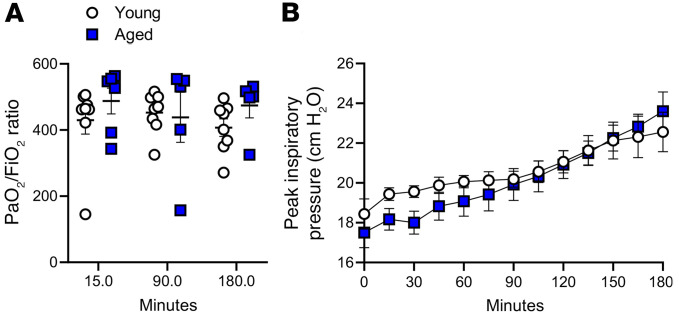
Effect of age on the physiological respiratory function during mechanical ventilation (20 mL/kg for 3 hours). (**A**) Throughout the time course of mechanical ventilation, no significant differences were observed in the PaO_2_/FiO_2_ ratio between the young and aged animals. (**B**) No significant differences were observed in peak inspiratory pressure. *n* = 8 (young) and *n* = 6 (aged). Unpaired *t* test and repeated-measures 1-way ANOVA were used.

To examine the effect of age on the pulmonary response to mechanical ventilation, both young and aged mice were ventilated for 3 hours prior to collecting tissues for analysis. Ventilation parameters were set based on weight, and in general, aged mice were significantly heavier than young mice (37.88 ± 1.88 g versus 29.97 ± 2.01 g, respectively). No significant differences were observed between young and aged animals in terms of gas exchange or lung function, as indicated by the ratio of partial pressure of oxygen in arterial blood to the fraction of inspiratory oxygen (PaO_2_/FiO_2_) or peak inspiratory pressure, respectively (Figure 1, A and B).

**Figure 2:**
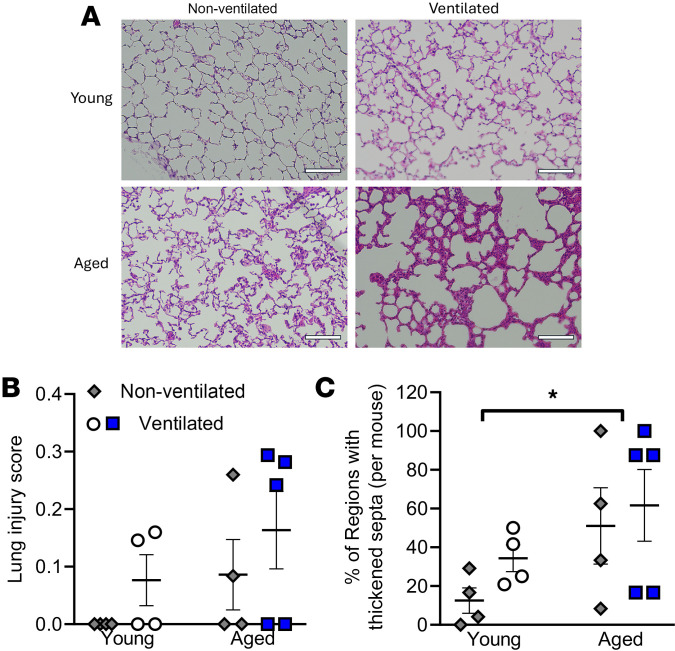
Lung histology, lung injury score, and alveolar septal thickness analysis of young and aged nonventilated and ventilated mice. (**A**) Representative images of lung histology show that, relative to young nonventilated lungs, ventilated and aged lungs, independently and in combination, appear to have thickened alveolar walls. (**B**) Although not significant, a trend toward increased injury was observed with ventilation and with aging. (**C**) A significant increase in the proportion of alveolar regions with thickened septa was observed with aging. *n* = 4–5; *P < 0.05; 2-way ANOVA with Tukey’s test. Scale bar: 100 μm.

To complement the physiological analyses, histological assessment of the lungs was performed, with representative images shown in Figure 2A. Lung injury scoring was performed by a blinded pathologist according to the American Thoracic Society report on measurements of experimental acute lung injury, which assesses alveolar space neutrophils, interstitial neutrophils, hyaline membrane formation, proteinaceous debris, and septal thickening (60). Although not significant, trends toward greater overall lung injury scores were observed with ventilation and aging (Figure 2B). Of the 5 criteria used for lung injury assessment, one of the primary contributors to the increased scores observed with ventilation and aging was septal thickening. Further analysis examining differences in septal thickness revealed that, although mean septal width was not significantly different across all groups, a larger number of regions were found to be thickened following ventilation, as well as with age (Supplemental Figure 1; supplemental material available online with this article; https://doi.org/10.1172/jci.insight.185834DS1). When directly assessing the percentage of regions with thickened septa, a significant increase was observed with age, both under control and ventilation conditions (Figure 2C).

### scRNA-Seq cell identification.

To provide insight into the effect of aging and mechanical ventilation on specific pulmonary cell types, scRNA-Seq analysis was performed (Supplemental Figure 2). Following quality control filtering steps, 10,172 cells were retained (young nonventilated, 2,538; young ventilated, 2,977; aged nonventilated, 2,483; aged ventilated, 2,174). Additional steps of normalization, scaling, and calculation of appropriate principal components identified a total of 19 distinct clusters of cells (Supplemental Figure 2A). Cluster annotation identified several pulmonary tissue cell types, including alveolar epithelial type I and II cells, capillary type I and type II cells, and inflammatory cell types, such as alveolar macrophages and classical monocytes (Supplemental Figure 2A). Key marker genes utilized to identify specific clusters demonstrated specificity with minimal overlap between clusters (Supplemental Figure 2B). These scRNA-Seq data were then subsequently used to assess the responses of specific cell types that are known to be relevant in the pathophysiological processes that take place during mechanical ventilation.

### Effect of age on pulmonary surfactant during mechanical ventilation.

**Figure 3:**
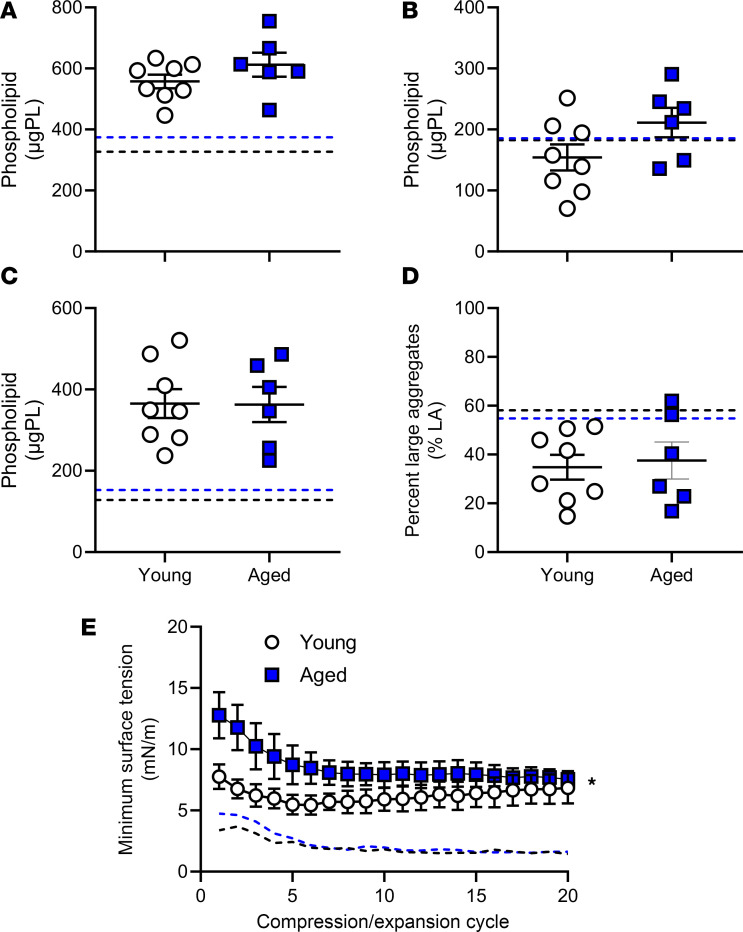
Effect of aging during mechanical ventilation on surfactant pool size and surfactant function. Values for nonventilated animals are included for reference from a previous study (52) and are indicated as dashed lines (black for young; blue for aged). (**A**–**D**) No significant differences were observed in total surfactant abundance (**A**), large (**B**) or small (**C**) aggregates, or percentage of large aggregates relative to total surfactant (**D**). There appeared to be an increase in minimum surface tension of surfactant in both young and aged mice when compared with historical controls (52). (**E**) The minimum surface tension of surfactant was significantly increased in aged versus young mice following ventilation. *n* = 5–8; *P < 0.05; unpaired *t* test and repeated-measures 1-way ANOVA.

To examine the effect of age on pulmonary surfactant during mechanical ventilation, surfactant isolated from young and aged mice was analyzed (Figure 3). While surfactant from young and aged nonventilated controls was not included in the analysis, values from a previous study with similar young and aged mice that were not ventilated have been included for comparative purposes (Figure 3) (52). No differences in surfactant amounts or composition (i.e., large versus small aggregates) were noted between young and aged mice following ventilation (Figure 3, A–D). There was, however, the suggestion that mechanical ventilation led to higher amounts of surfactant, specifically the small aggregate forms, in both young and aged mice when compared with previous findings (52) (Figure 3, A and C). Functional analysis of the surfactant also revealed that ventilated animals appeared to have higher minimum surface tension as compared with the previously published values (52) (Figure 3E). Moreover, aged ventilated mice also had a significantly higher minimum surface tension versus young ventilated mice (Figure 3E).

**Figure 4:**
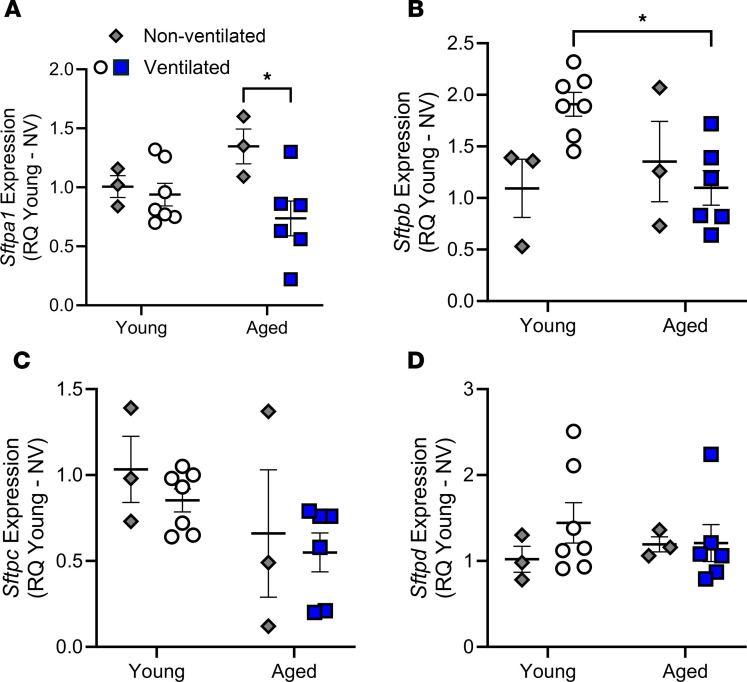
Effect of age and mechanical ventilation on SFTP expression. Following mechanical ventilation (20 mL/kg for 3 hours), the expression of *Sftpa1* (**A**), *Sftpb* (**B**), *Sftpc* (**C**), and *Sftpd* (**D**) was assessed by qPCR in whole lung tissue of young and aged mice. A significant reduction in *Sftpa1* was observed in aged ventilated compared with aged nonventilated mice (**A**). A significant decrease in *Sftpb* was also observed in the aged ventilated mice compared with young ventilated mice (**B**). No differences were observed in *Sftpc* (**C**) or *Sftpd* (**D**). *n* = 3–7; *P < 0.05; 2-way ANOVA with Tukey’s test.

Analysis of surfactant protein (SFTP) expression by quantitative PCR (qPCR) revealed that aged ventilated mice had significantly decreased *Sftpa1* relative to aged nonventilated mice (Figure 4A). The aged ventilated mice also had a significant decrease in *Sftpb* compared with young ventilated animals (Figure 4B). No significant differences were observed in *Sftpc* or *Sftpd* between young and aged mice (Figure 4, C and D). Additionally, mechanical ventilation did not appear to significantly affect the expression of SFTPs in young mice.

**Figure 5:**
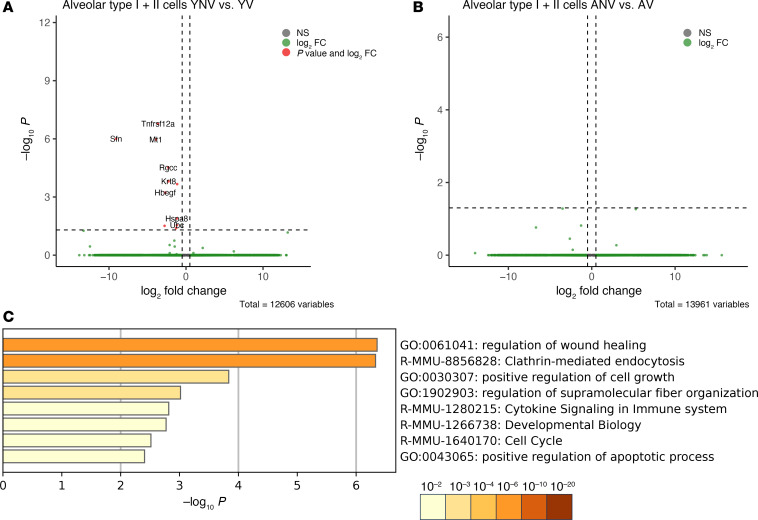
Effect of age on the transcriptomic response to mechanical ventilation in alveolar type I + II cells. (**A** and **B**) The volcano plot reveals only a few significantly differentially expressed genes in the young animals (**A**) and no significantly differentially expressed genes in the aged animals (**B**) following mechanical ventilation. (**C**) The heatmap highlights a few enriched pathways associated with cell activation that are found exclusively in the young animals following mechanical ventilation. Datasets were derived from *n* = 3 animals pooled per group. YNV, young nonventilated; YV, young ventilated; ANV, aged nonventilated; AV, aged ventilated.

To assess the effect of age and mechanical ventilation on the alveolar type II cells responsible for production of pulmonary surfactant, differential gene expression analysis was performed on the scRNA-Seq dataset. Similar to other studies, annotation of our scRNA-Seq clusters yielded a group of alveolar type I + II cells (Supplemental Figure 2), and it was this group that was analyzed (61). No differences in gene expression were identified between young and aged mice under basal conditions (data not shown). Furthermore, when evaluating differences in gene expression between young and aged mice following mechanical ventilation, minimal differences were again observed. Just 11 genes were identified as significantly differentially expressed in the alveolar type I + II cells between the young nonventilated and young ventilated mice (Figure 5A), with zero genes being identified in the aged mice (Figure 5B). Based on these genes, a limited number of pathways associated with cell activation and wound healing were found to be enriched in the young animals compared with the aged animals (Figure 5C).

### Effect of age on pulmonary inflammation during mechanical ventilation.

**Figure 6:**
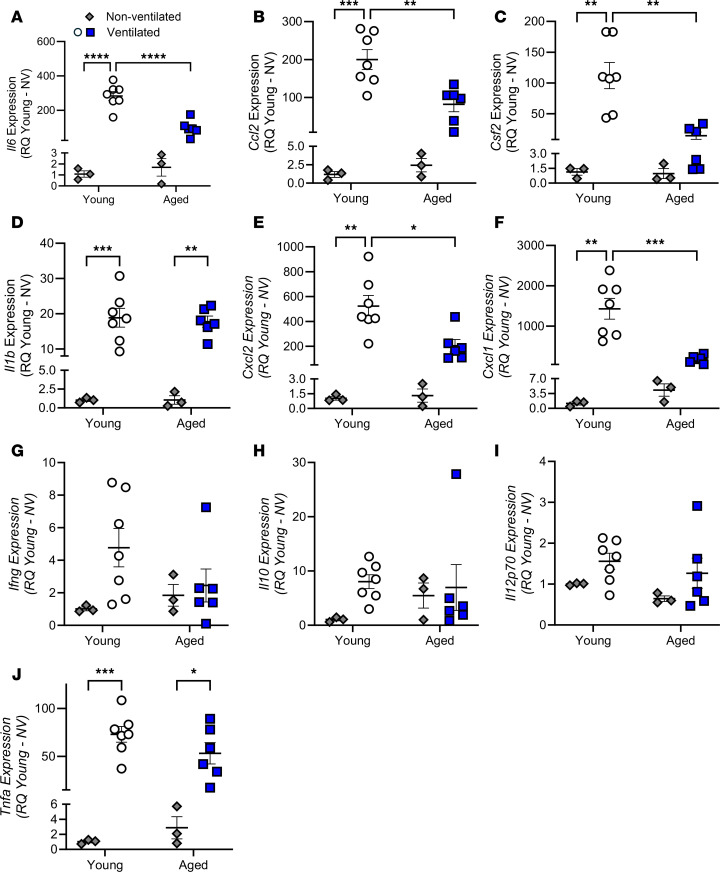
Effect of age and mechanical ventilation (20 mL/kg for 3 hours) on expression of inflammatory cytokines. (**A**–**J**) The expression levels of *Il6* (**A**), *Ccl2* (**B**), *Csf2* (**C**), *Il1b* (**D**), *Cxcl2* (**E**), *Cxcl1* (**F**), *Ifng* (**G**), *Il10* (**H**), *Il12p70* (**I**), and *Tnfa* (**J**) were assessed by qPCR in whole lung tissue from young and aged mice. *n* = 3–7. Significance is shown as *P < 0.05; **P < 0.01; ***P < 0.001; ****P < 0.0001; 2-way ANOVA with Tukey’s test.

**Figure 7:**
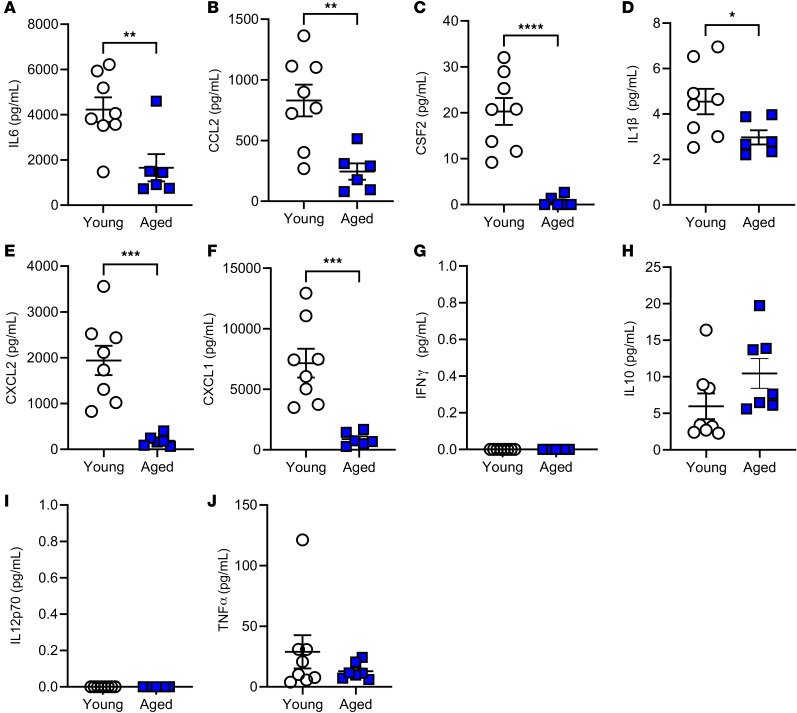
Effect of age during mechanical ventilation on inflammatory cytokine protein abundance within the serum. Following mechanical ventilation (20 mL/kg for 3 hours), multiplex analysis was performed to assess the abundance of IL6 (**A**), CCL2 (**B**), CSF2 (**C**), ILIβ (**D**), CXCL2 (**E**), CXCL1 (**F**), IFNγ (**G**), IL-10 (**H**), IL-12P70 (**I**), and TNFα (**J**) within the serum. *n* = 8 (young) and *n* = 6 (aged). Significance is shown as *P < 0.05; **P < 0.01; ***P < 0.001; ****P < 0.0001; unpaired *t* test.

**Figure 8:**
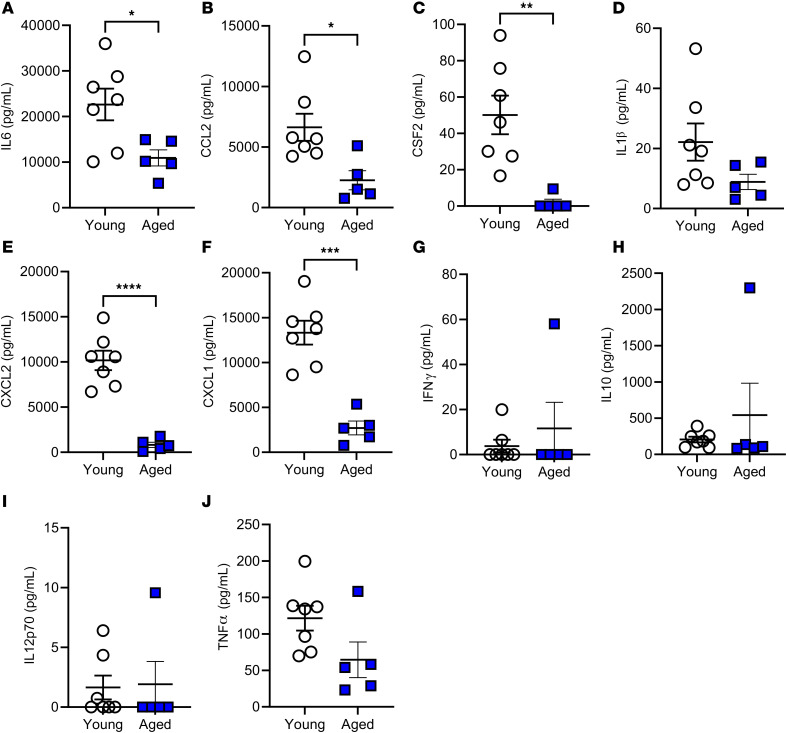
Effect of age during mechanical ventilation on inflammatory cytokine protein abundance within the serum. Following mechanical ventilation (20 mL/kg for 3 hours), multiplex analysis was performed to assess the abundance of IL6 (**A**), CCL2 (**B**), CSF2 (**C**), IL-1β (**D**), CXCL2 (**E**), CXCL1 (**F**), IFNγ (**G**), IL-10 (**H**), IL-12P70 (**I**), and TNFα (**J**) within the serum. *n* = 7 (young) and *n* = 5 (aged). Significance is shown as *P < 0.05; **P < 0.01; ***P < 0.001; ****P < 0.0001; unpaired *t* test.

To explore the effect of age on the inflammatory response during mechanical ventilation, inflammatory cytokines were assessed in the lung tissue by qPCR (Figure 6) as well as within the lung lavage fluid (Figure 7) and serum (Figure 8) by multiplex analysis. Mechanical ventilation resulted in a significant increase in the expression of *Il6*, *Ccl2*, *Csf2*, *Il1b*, *Cxcl2*, *Cxcl1*, and *Tnfa* in the young mice following ventilation; however, only *Il1b* and *Tnfa* were significantly increased in aged mice following ventilation (Figure 6). The aged mice also had significantly decreased expression of *Il6*, *Ccl2*, *Csf2*, *Cxcl2*, and *Cxcl1* versus young mice following mechanical ventilation (Figure 6). Notably, no statistical differences were noted between the young and aged nonventilated mice. Based on this, cytokine analyses of the lavage and serum by multiplex analysis were carried out with comparisons between only the young and aged ventilated groups. Similar to cytokine mRNA expression, measurement of the cytokine protein levels within the lavage fluid from the ventilated animals revealed significantly decreased IL-6, CCL2, CSF2, IL-1B, CXCL2, and CXCL1 in aged ventilated versus young ventilated mice (Figure 7). Analysis of cytokine protein abundance within the serum revealed the same trends, with IL-6, CCL2, CSF2, CXCL2, and CXCL1 significantly decreased in the aged versus young mice following ventilation (Figure 8). Cell recovery from the lavage fluid from this cohort of animals was also performed, and cell counts were 3.9 × 10^5^ ± 0.55 × 10^5^ cells for aged ventilated animals and 1.8 × 10^5^ ± 0.29 × 10^5^ cells for young ventilated animals. Cell differentials revealed that over 99% of these cells were macrophages in both groups.

**Figure 9:**
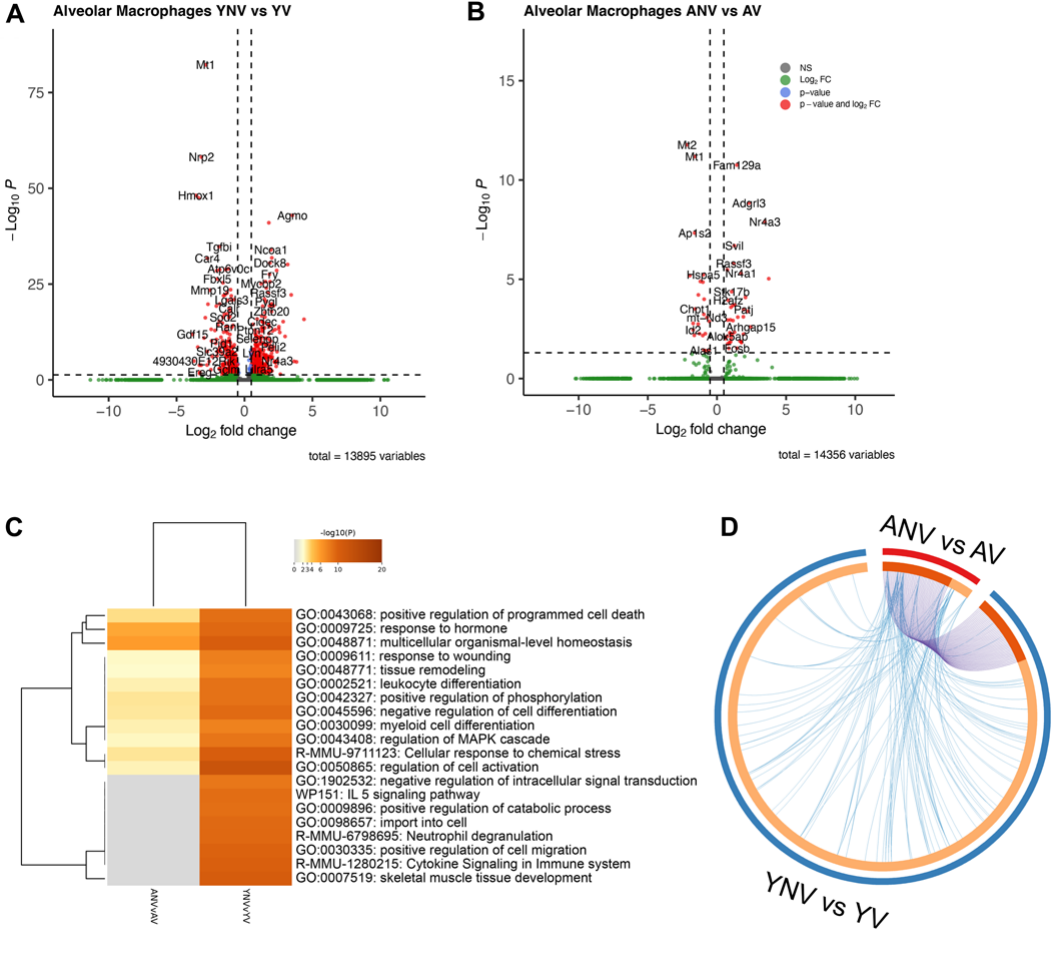
Effect of age on the transcriptomic response to mechanical ventilation in alveolar macrophages. (**A** and **B**) The volcano plots reveal many significantly differentially expressed genes in macrophages from the young animals following mechanical ventilation (**A**), with far fewer genes observed in the macrophages from the aged animals (**B**). (**C**) The heatmap highlighting enriched pathway terms regulated by differentially expressed genes in the alveolar macrophages demonstrates greater enrichment of inflammatory and activation pathways in the young mice exposed to mechanical ventilation, which is reduced in the aged mice. (**D**) The Circos plot displaying the degree of overlap of functional pathways between the young and aged animals in response to mechanical ventilation reveals high overlap of genes (blue lines) and pathways (purple lines) in the aged animals, as well as an abundance of genes and pathways exclusive to young animals. YNV, young nonventilated; YV, young ventilated; ANV, aged nonventilated; AV, aged ventilated.

**Figure 10:**
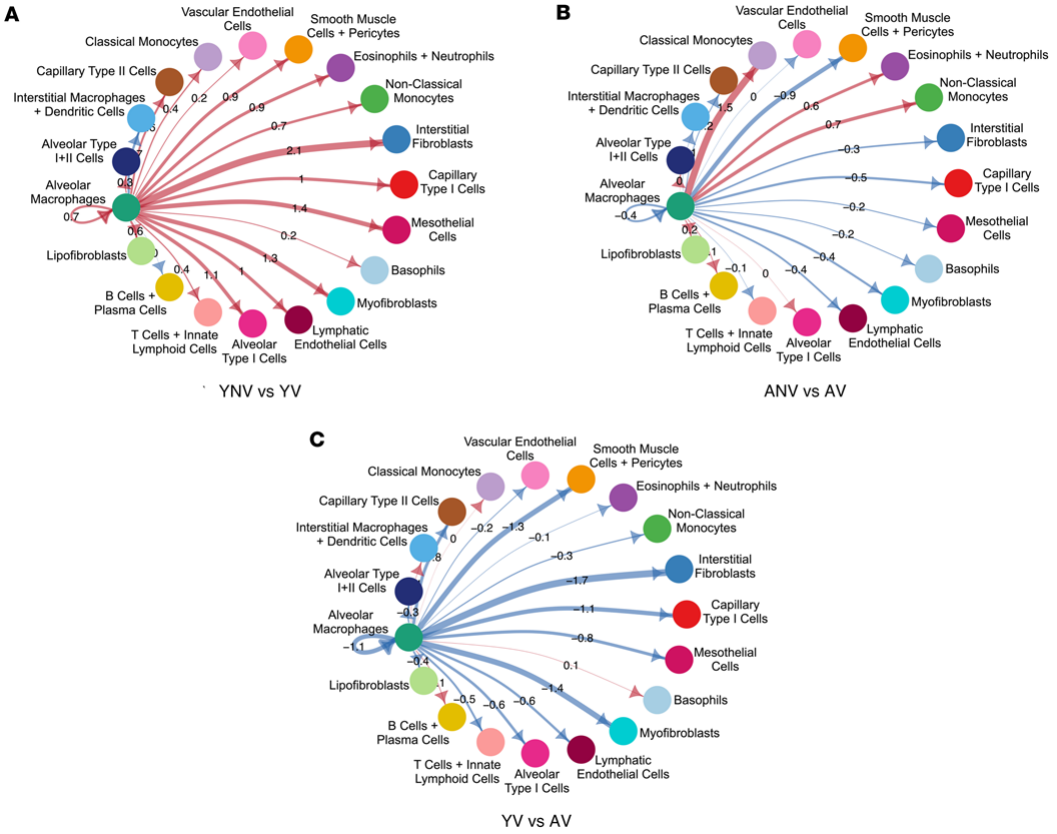
The effect of ventilation and aging on inferred secreted signaling originating from alveolar macrophages. (**A**) Inferred cell-cell communication analysis of secreted signaling pathways reveals an increase in communication from the alveolar macrophages to all other cell types in the young group following exposure to mechanical ventilation, as indicated by the red lines. (**B**) In contrast, the alveolar macrophages from aged animals exhibited a generally decreased response following mechanical ventilation, as indicated by the blue lines. (**C**) Comparison of the ventilated animals directly reveals an overall blunted response in the aged animals compared with young animals. Datasets were derived from *n* = 3 animals pooled per group. For circle diagrams (**A**–**C**), red lines indicate more signaling in second group relative to first group; blue lines indicate less signaling in second group relative to first group. YNV, young nonventilated; YV, young ventilated; ANV, aged nonventilated; AV, aged ventilated.

One of the primary cell types thought to initiate and propagate the inflammatory response during mechanical ventilation is the alveolar macrophage (21, 22). To understand how aging affects the response of alveolar macrophages during ventilation, differential gene expression analysis comparing alveolar macrophages from nonventilated versus ventilated young and aged mice was performed using the scRNA-Seq dataset. Under basal conditions, 34 genes were differentially expressed in the young nonventilated compared with aged nonventilated, most of which were upregulated in the aged (Supplemental Figure 3A). When performing gene ontology (GO) analysis, pathways associated with cell activation, cell adhesion, and inflammation were found to be differentially enriched between the 2 groups (Supplemental Figure 3B). Comparisons were then subsequently performed to examine the response to mechanical ventilation. A total of 536 genes were identified as differentially expressed between alveolar macrophages from the young nonventilated and ventilated mice (Figure 9A); however, only 61 genes were found to be differentially expressed in the alveolar macrophages from aged nonventilated versus ventilated mice (Figure 9B). While there were many pathways associated with cell activation that were shared between the young and aged mice, these pathways were more significantly enriched in the young mice (Figure 9C). There were also additional inflammatory pathways, including “IL5 signaling,” “cytokine signaling in immune system,” and “neutrophil degranulation” that were exclusively found in the young mice following mechanical ventilation (Figure 9C). Analysis of the circos plot demonstrated that the majority of genes differentially expressed in the alveolar macrophages from aged mice were similar to those differentially expressed in the young mice, as indicated by the purple lines (Figure 9D). There were, however, several genes and pathways that were exclusively enriched in the young mice (Figure 9D). Additional analysis was performed using CellChat, specifically focusing on communication patterns associated with secreted signaling originating from the alveolar macrophages. The CellChat analysis revealed an increase in the strength of secreted signaling from the alveolar macrophages to almost all other cell types in the young ventilated animals compared with the young nonventilated (Figure 10A). In contrast, there was a decrease in the strength of signaling found in the aged ventilated versus the aged nonventilated mice (Figure 10B). When directly comparing the aged ventilated group with the young ventilated group, there was also an overall blunted response in the strength of secreted signaling from the alveolar macrophages to almost all other cell types in the alveolar macrophages from aged mice (Figure 10C).

### Effect of age on pulmonary microvascular barrier function during mechanical ventilation.

**Figure 11:**
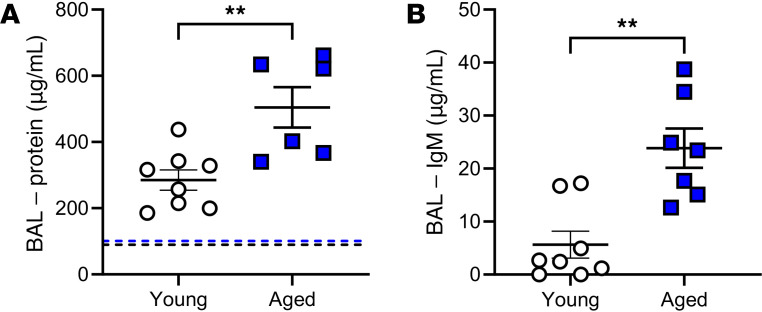
Effect of aging during mechanical ventilation on protein leak within the lung. Values for nonventilated animals are shown as reference from a previous study for the protein leak assessment (52) and are indicated as dashed lines (black for young; blue for aged). (**A** and **B**) Compared with young ventilated animals, aged ventilated animals exhibited a significant increase in protein (**A**) and IgM (**B**) in the bronchoalveolar lavage fluid. *n* = 8 (young) and *n* = 6–7 (aged). **P < 0.01; unpaired *t* test.

A key pathophysiological process associated with VILI is pulmonary microvascular leak contributing to the accumulation of protein-rich edema fluid. The effect of age on microvascular leak following mechanical ventilation was assessed by analysis of total lavage protein and lavage IgM levels, both of which have been shown to correlate with wet/dry ratios and lung edema (62) (Figure 11). Comparison of total protein within the lavage fluid of young and aged mice following ventilation to data from previously published work suggests that ventilation appears to increase total lavage protein in both groups (52) (Figure 11A). Furthermore, following ventilation, total protein was also significantly increased in aged versus young mice (Figure 11A). Similarly, total IgM was significantly increased in lavage fluid from aged ventilated mice compared with young ventilated mice (Figure 11B).

To begin examining potential cellular mechanisms involved in the augmented pulmonary microvascular leak observed in aged animals following mechanical ventilation, differential gene expression analysis was conducted on the capillary cell populations identified in the scRNA-Seq dataset. Like other studies, 2 capillary populations were identified based on known marker genes used in the literature; the first was the capillary type I cells, also commonly referred to as aerocyte capillary cells, which are specialized for gas exchange, and the second population was the capillary type II cells, also referred to as general capillary cells, which function as a progenitor-like cell involved in repair (63–65). Under basal conditions in the capillary type I cells, 33 genes were upregulated in the young nonventilated animals, while 31 genes were upregulated in the aged nonventilated animals (Supplemental Figure 4A); this led to pathways associated with the actin cytoskeleton, cytoplasmic ribosomal proteins, and cytokine signaling that were differentially enriched between the 2 groups (Supplemental Figure 4B). Meanwhile, under basal conditions, only 1 gene was differentially expressed in the capillary type II cells between young and aged animals, suggesting no baseline differences (Supplemental Figure 4C).

**Figure 12:**
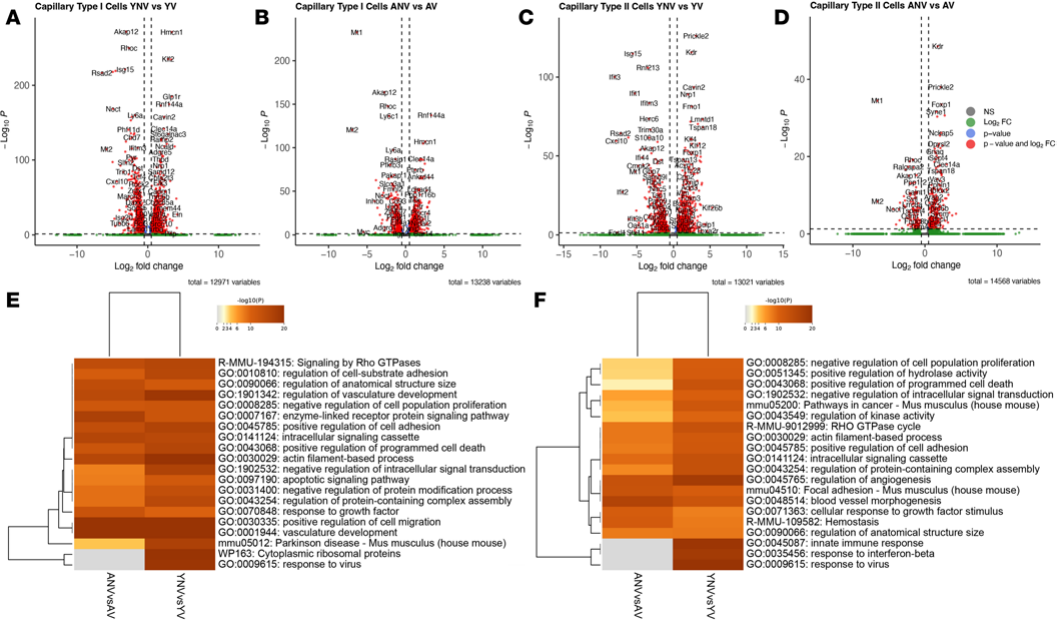
Effect of age on the transcriptomic response to mechanical ventilation in capillary type I and capillary type II cells. (**A** and **B**) The volcano plots demonstrate several significantly differentially expressed genes in the capillary type I cells following mechanical ventilation in both young (**A**) and aged animals (**B**). (**C** and **D**) The same trend was observed for capillary type II cells. (**E** and **F**) The heatmaps highlight several enriched pathways associated with vessel development and cell-cell adhesion in both the young and aged mice in both capillary populations. Datasets were derived from *n* = 3 animals. YNV, young nonventilated; YV, young ventilated; ANV, aged nonventilated; AV, aged ventilated.

**Figure 13:**
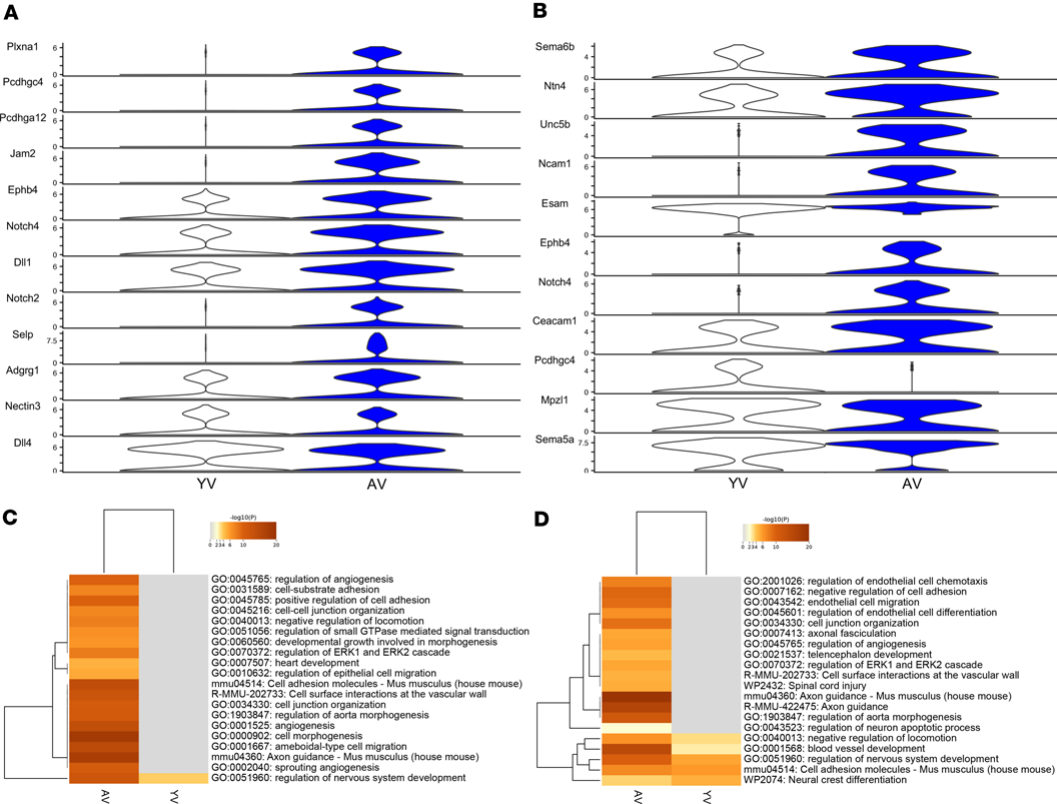
The effect of aging on inferred cell-cell contact autocrine signaling from the capillary cells during mechanical ventilation. (**A** and **B**) The CellChat violin plots of inferred cell-cell contact signaling pathways reveals increased expression of several ligands and receptors associated with cell-cell adhesion in the aged ventilated compared with young ventilated mice in both capillary type I (**A**) and type II (**B**) cells (ligands and receptors shown are the most differentially enriched). (**C** and **D**) Gene ontology analysis using all differentially enriched ligands and receptors revealed that the aged ventilated animals exhibited a robust enrichment in pathways associated with cell-cell adhesion, cell junction organization, angiogenesis, and blood vessel development in both capillary type I (**C**) and type II (**D**) cells. Datasets were derived from *n* = 3 animals. YNV, young nonventilated; YV, young ventilated; ANV, aged nonventilated; AV, aged ventilated.

When assessing the response to mechanical ventilation, a total of 1,344 genes was identified as differentially expressed in the capillary type I cells from the young animals (Figure 12A), and 953 genes were differentially expressed in the aged animals (Figure 12B). Similarly, in the capillary type II cells, 934 genes were differentially expressed in the young animals (Figure 12C), with 341 genes differentially expressed in the aged animals (Figure 12D). GO analysis of the capillary type I and type II cells demonstrated that similar pathways were enriched in both the young and aged animals, including those related to vessel development, angiogenesis, and cell-cell adhesion (Figure 12, E and F). CellChat analysis was performed to specifically examine and enrich for autocrine cell-cell contact pathways at the level of the capillary cell populations and to directly compare the young and aged ventilated animals. Interestingly, compared with young ventilated, the aged ventilated mice exhibited upregulation of many genes associated with cell-cell adhesion within both capillary populations. In particular, there were 39 genes enriched in the capillary type I cells from the aged ventilated animals compared with just 5 genes that were enriched in the young ventilated animals. Similarly, there were 28 genes enriched in the capillary type II cells from the aged ventilated animals compared with 10 genes enriched in the young ventilated animals. The most differentially enriched genes are shown in Figure 13, A and B. Key cell-cell contact gene signals identified as elevated in aged ventilated mice included Junction Adhesion Molecule, Endothelial Cell-Specific Adhesion Molecule, Nectin3, and Protocadherins, all of which are directly involved in endothelial cell-cell adhesion and maintenance of vascular barrier function (Figure 13, A and B). This was further highlighted when performing GO analysis using all differentially enriched cell-cell contact genes; compared with the young ventilated animals, the capillary cells from the aged ventilated animals exhibited robust enrichment of pathways associated with cell-cell adhesion, cell junction organization, and angiogenesis (Figure 13, C and D).

**Figure 14:**
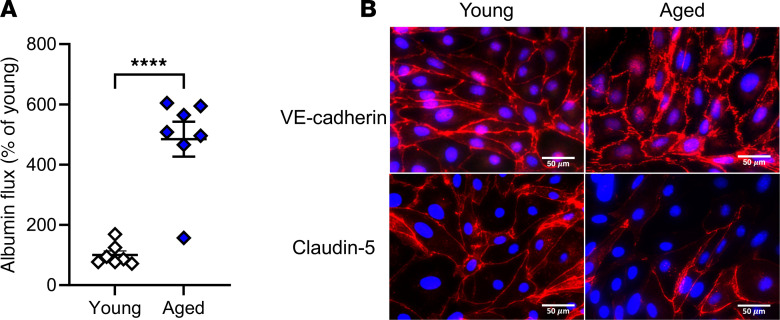
Assessment of endothelial barrier integrity in isolated PMVEC from young and aged animals, grown to confluent monolayers. (**A**) Compared with young PMVEC, aged PMVEC had a significant 484% increase in monolayer permeability, as assessed by albumin flux in the transwell system. (**B**) Staining of adherens junctional protein, VE-cadherin, and tight junctional protein claudin-5 revealed disrupted localization in the PMVEC monolayers from aged mice compared with young mice; above are representative images from *n* = 5 experiments. For albumin flux experiments, *n* = 7. Scale bar: 50 μm. ****P < 0.0001; unpaired *t* test.

To assess whether the defects observed in aged versus young pulmonary microvascular endothelial cells (PMVEC) were due specifically to changes within these cells versus within the microenvironment, PMVEC were isolated and cultured in vitro to assess their ability to form a barrier (Figure 14). Analysis of barrier function through measurement of albumin flux across a transwell system revealed a significant increase in permeability in PMVEC isolated from aged versus young mice under basal conditions (Figure 14A). Moreover, immunocytochemical analysis of both VE-cadherin (adherens junctions) and claudin-5 (tight junctions) identified disrupted localization along the periphery of PMVEC from aged mice under basal conditions; this was in stark contrast to continuous and smooth signaling present in the PMVEC from young mice (Figure 14B).

## Discussion

The primary goal of our study was to investigate the effect of aging on the pulmonary responses during mechanical ventilation. Overall, the hypothesis that aging leads to alterations in surfactant function, inflammatory signaling, and microvascular permeability within the lung during mechanical ventilation was supported by the findings, specifically the impaired surfactant function, diminished inflammation, and augmented microvascular leak observed in aged versus young mice. Experimentally, we utilized mice of 2–3 months of age and mice that were 22 months old. These ages are comparable with approximately 20 and 65 years old in humans. Mice were ventilated at a high tidal volume of 20 mL/kg, which is higher than the volumes used in clinical practice in humans. Although lung mechanics were not measured in the current study, the high tidal volume appeared to be well tolerated over 3 hours in both age groups, as illustrated by our oxygenation data, peak inspiratory pressures, and histological assessments, consistent with the literature (66). It was rationalized that such a high tidal volume would lead to overstretching of the lung and reflect the effect of mechanical ventilation over a relatively short period of time on injured lungs, such as those found in patients with ARDS. This is a strategy that has been extensively employed in many studies using healthy animals to mimic injury that may occur over prolonged periods with lower tidal volumes in humans, where an underlying injury is present (13, 14, 16). Given that mechanical ventilation is a common therapeutic used in a variety of respiratory diseases where oxygenation is impaired, we opted to not induce an initial insult or infection to allow us to examine direct mechanisms associated with aging and lung injury induced by mechanical ventilation. For the outcomes, we combined traditional biochemical methods with scRNA-Seq to provide a comprehensive assessment of various pathophysiologies in young and aged mechanically ventilated mice, which, to our knowledge, allowed for the generation of novel data. Overall, these experimental conditions allowed us to test our hypothesis and potentially extrapolate our data to the clinical situation in humans.

The main, unanticipated outcome of our study was the differential effect of aging on the 3 pathophysiological mechanisms. In general, when examining the literature, increased severity of lung injury is associated with more pronounced surfactant dysfunction, increased inflammation, and greater vascular leak and edema formation (1, 30, 36, 40, 67–69). Based on the fact that the elderly have worse outcomes from ARDS requiring mechanical ventilation, it was therefore anticipated that each of these pathophysiological processes would be more severe in our mechanically ventilated aged mice. However, the pattern of the observed changes was distinct for each of these processes. First, the inflammatory response was significantly lower in aged mice compared with young. Second, no differences were observed in the surfactant pools due to ventilation, but the gene expression of *Sftpa1* (relative to nonventilated) and *Sftpb* (relative to young ventilated) were decreased in aged ventilated animals. Furthermore, surfactant dysfunction was exacerbated in the aged mice following ventilation, which was associated with minimal differences observed at the level of the transcriptome of the surfactant producing alveolar type II cells. Lastly, vascular protein leak was significantly increased in aged versus young mice. While lung edema was not directly assessed in this work, other studies have demonstrated that injurious ventilation leads to increased vascular protein leak within the lung, which subsequently can promote pulmonary edema formation (8, 70). Additionally, protein and immunoglobulin levels within the bronchoalveolar lavage fluid have been positively correlated with lung wet/dry ratios following mechanical ventilation (62). It has been extensively demonstrated in the literature that increased pulmonary vascular leak and lung edema can contribute to inactivation of surfactant (33, 44–46). This, possibly in conjunction with alterations in SFTP expression, is likely what mediated the surfactant dysfunction in our aged animals following mechanical ventilation, despite the absence of major changes to surfactant amounts or to the surfactant-producing alveolar type II cells. Notably, these patterns were observed at both the biochemical level and within our scRNA-Seq data. Collectively, this supports the conclusion that aging leads to increased vascular leak following mechanical ventilation, independent of an augmented inflammatory response or alterations to the surfactant system.

The above interpretation that the surfactant system and inflammation did not affect permeability implies that the altered vascular response to ventilation is due to other age-related processes. Both examination of isolated PMVEC and examination of our scRNA-Seq data indicate that cell-cell adhesion between capillary endothelial cells may be involved. First, we observed enhanced protein leak in isolated aged PMVECs as compared with young cells that was associated with impairments in developing appropriate cell-cell adherens and tight junctions. Second, whereas the scRNA-Seq dataset showed many similar pathways that were enriched between the young and aged animals following ventilation, homing in on the specific cell-cell contact pathways showed an overall increase in enrichment of many cell-cell adhesion molecules in aged animals compared with young. While initially counterintuitive, these findings may suggest that the capillary endothelial cells in the aged ventilated animals are more readily activated and susceptible to damage and are subsequently upregulating genes involved in cell-cell adhesion in a compensatory manner to promote vascular barrier repair following mechanical ventilation. Therefore, our second conclusion is that an age-dependent predisposition to impaired endothelial barrier formation, due to impaired cell-cell junctions, causes an increase in vascular leak due to mechanical ventilation.

Although our data on inflammation do not provide insight into a potential susceptibility of the elderly to mechanical ventilation, we observed a general decrease in inflammatory mediators in aged animals. This finding was supported by mRNA and protein data for a selection of mediators, indicating a broad and robust difference in inflammation, rather than an effect related to a specific mediator — a concept that was supported by the scRNA-Seq alveolar macrophage data. Certainly, we are not the first to see a blunted inflammatory response with age. In fact, an age-dependent blunted inflammatory response to stretch at acute time points has been shown in an in vitro system (71), supporting our in vivo findings. One suggested mechanism to explain this is the idea of inflammaging: the age-related increase in proinflammatory cytokine levels in blood and tissues under baseline conditions (48, 51), followed by immune exhaustion or immunosenescence in response to a subsequent injury (49, 50). Taken together, these data suggest an overall suppressed inflammatory response in the aged mouse lungs compared with young when exposed to mechanical ventilation, which is likely driven by blunted activation of the alveolar macrophages. While other studies have identified increased vascular leak with age following mechanical ventilation, our study was the first to our knowledge to identify this response in the absence of elevated inflammation (13–16).

Interestingly, additional in vivo studies assessing the effects of aging during mechanical ventilation, with or without an initial insult, have found enhanced inflammation with age, as assessed by proinflammatory cytokine quantification in the serum and within the lung, as well as neutrophil infiltration within the airspaces (13–15, 17). Potential reasons for this discrepancy in inflammatory signaling with age during mechanical ventilation may have been due to different animal models and ages, different time points, and different ventilation parameters. From our data, basal scRNA-Seq differences between the young and aged nonventilated mice indicated an overall increase in activation of the alveolar macrophages, which is supportive of inflammaging. A subsequent overall blunted mechanical ventilation response with age with respect to cytokine signaling, immune activation, and cell-cell communication pathways associated with secreted signaling from alveolar macrophages, further supports the mechanism of immune exhaustion.

Another relevant mechanism to consider is the beneficial role of inflammation and how this is affected with age. In particular, acute inflammation has been extensively demonstrated to promote the initiation of wound healing (72–75). This includes recruitment of cells like macrophages to remove neutrophils as well as promote angiogenesis, fibroblast proliferation, and extracellular matrix production (76). Consequently, alveolar macrophages have been shown to promote lung repair following injury (77, 78). Our data may suggest a role for aging in attenuating alveolar macrophage–mediated acute inflammation, which may be affecting potential reparative mechanisms following mechanical ventilation. However, given the highly acute nature of this study, it is challenging to parse out the contribution of reparative or injurious inflammatory signaling.

Speculating on the implications of our findings toward the human setting, it appears that, at least of the 3 pathophysiological pathways explored in this study, the main process that may lead to increased susceptibility to greater morbidity during mechanical ventilation in the elderly is vascular permeability. There are currently no treatments that mitigate vascular permeability within the lung; however, augmented vascular permeability has been identified in many serious diseases, such as traumatic injury, sepsis, and shock (79–83). The prevalence of vascular permeability as a common pathophysiological process in these diseases has led to ongoing research into potential prophylactic and therapeutic treatments with limited success (83), leading to the potential of future treatment to rescue microvascular barrier function within the lungs of aged individuals undergoing mechanical ventilation.

While our study utilizes a number of complementary approaches to assess the effect of age on mechanical ventilation in vivo, there are several limitations, such as the use of only male mice and a high, injurious tidal volume. Additionally, our model of mechanical ventilation only utilized a single time point of 3 hours, which is considered an acute injury and may not capture the ongoing propagation of injury that occurs with VILI over extended periods of time. Finally, since our focus was on the effect of mechanical ventilation alone, there was no initial primary injury or infection. While this does not completely recapitulate the clinical scenario, it enabled a focus on the specific mechanisms of injury associated with stretch and the overdistension that occurs with mechanical ventilation. Future studies will be focused on addressing these limitations, especially focusing on the role of biological sex. Additionally, future work will also begin to explore the role of other cell types that may be involved in the altered permeability, such as fibroblasts, monocytes, pericytes, and alveolar type I cells. While not within the scope of the current study, assessing the effects of age on the response of the alveolar type I cells to mechanical ventilation will be particularly crucial, as they are also known to be implicated during VILI, leading to loss of epithelial barrier function (26).

In summary, our study aimed to elucidate the effect of aging on pulmonary responses during mechanical ventilation. The findings support the hypothesis that aging alters these responses, particularly highlighting impaired surfactant function, reduced inflammation, and increased microvascular leak in aged mice compared with young mice. Utilizing both traditional biochemical methods and scRNA-Seq, we provided a comprehensive assessment of these pathophysiological changes. The data suggest that aging leads to increased vascular leak independent of a hyperinflammatory response or robust alterations in the surfactant system. We suggest that age-related changes in PMVECs, specifically in cell-cell adhesion mechanisms, contribute to the increased vascular leak observed in aged mice. These findings offer insights into the cellular and molecular mechanisms underpinning age-related susceptibility to lung injury during mechanical ventilation and may inform future therapeutic strategies.

## Methods

### Sex as a biological variable.

The current study was performed exclusively in male mice for practical reasons related to maintenance of our aging mouse colony during the COVID-19 pandemic. The limitation of using only male mice is considered in the interpretation of the results. While clinical data suggest that females are more susceptible to injury from mechanical ventilation, it is unknown whether the findings are relevant for female aged mice (84).

### Animal model of aging.

C57BL/6 male mice from Charles River Laboratories were aged to 22 months under controlled conditions (standard light-dark cycles, temperature, humidity, and group-housing) within our institutional animal facility. Obesity has been shown to be associated with augmented proinflammatory responses, elevated risk of ARDS, changes in respiratory mechanics, alterations on pulmonary function during mechanical ventilation, and vascular damage (85–89). Consequently, the mice were fed a food-restricted diet with a gradual reduction to 70% ad libitum, to maintain body weight, as described previously (90).

### Model of mechanical ventilation.

Details of this model have been previously reported (91). Briefly, the young (2–3 months) and aged (22 months) mice were randomized to either the nonventilated control or mechanical ventilation group. Ventilated mice were anesthetized using ketamine hydrochloride (100 mg/kg) and medetomidine (50 mg/kg) and received 1.5 g of buprenorphine s.c. for analgesia. Arterial and venous vascular lines were set up for arterial blood gas measurements and administration of Ringer’s lactate fluid (0.2 mL/hour in the right jugular vein and 0.1 mL/hour in the carotid artery). A Harvard MidiVent mechanical ventilator (Harvard Apparatus) was connected via an endotracheal tube. Mice were ventilated for 3 hours at a respiratory rate of 150 breaths/min, a positive end expiratory pressure (PEEP) of 0 cm H_2_O, and a fraction of inspired oxygen (FiO_2_) of 1.0. The tidal volume was set at 20 mL/kg, to correct for variable body weights, up to a maximum of 0.6 mL, such that all the mice weighing 30 g and more received a tidal volume related to an estimate of lean body weight. Notably, a tidal volume of 20 mL/kg is considerably higher than clinically relevant values, but this volume was utilized to initiate tissue injury within the animals, with the consideration that mice are more resistant to VILI (66). Since preexisting damage would lead to areas of lower lung compliance that would promote overstretching of alveoli in healthier, higher compliance areas, and since this study was solely focused on mechanical ventilation in the absence of underlying injury, an initial insult was not conducted in the animals. Baseline physiological values were noted, and pulmonary blood gases and peak inspiratory pressure were measured throughout. Nonventilated controls were naive mice that did not undergo the above procedures.

### Lung fixation, H&E staining, and histological analyses.

Lungs were harvested and fixed by inflating to 25 cm H_2_O with 10% formalin. Following 24 hours, lungs were rinsed in PBS, dehydrated through an alcohol series, and embedded in paraffin wax for sectioning. H&E staining was carried out, and sections were imaged for analysis. Lung injury scores were calculated by a blinded pathologist according to the criteria outlined by the American Thoracic Society for assessment of experimental acute lung injury in animals (alveolar space neutrophils, interstitial neutrophils, hyaline membranes, proteinaceous debris, and septal thickening) (60). Alveolar septal measurements were taken in central and peripheral regions from 4 lobes of each mouse. Specifically, 3 measurements were taken around the center of the lobe near major airways, if present, as well as around the periphery of the lung lobes, defined as approximately 3 alveolar spaces from the pleura without septal thickening. Quantification of alveolar septal thickness was performed using QuPath. Values obtained from the young nonventilated animals were considered as the baseline, with septal measurements greater than 2 SDs from the mean of the baseline being considered as significantly thickened. Based on this, the proportion of alveolar regions with thickened septa from each mouse was assessed.

### Generation of single-cell suspensions from whole mouse lung.

A separate cohort of mice was euthanized via i.p. injection of pentobarbital sodium. The pulmonary vasculature was flushed with sterile PBS through the right ventricle of the heart. The lungs were removed, and the left lobe was isolated and minced to small pieces (approximately 1–3 mm^2^). The tissue pieces were transferred into a 15 mL falcon tube with 3mL RPMI media (Thermo Fisher Scientific, 21870076) containing 10% fetal bovine serum (FBS), 1% penicillin-streptomycin, and the digestion enzymes Liberase (400 μg/mL; MilliporeSigma, 5401127001) and DNase I (400 μg/mL; MilliporeSigma, DN25). The tube was placed on an end-to-end rotator and incubated at 37°C for 30 minutes. Following incubation, the enzymatic digestion was stopped using 100 mM EDTA at 37°C for 5 minutes. Dissociated cells in suspension were passed through a 100 µm cell strainer into a 50 mL Falcon tube with an additional 5 mL of ice-cold 2 mM EDTA in complete DMEM (DMEM supplemented with 10% FBS and penicillin-streptomycin). Centrifugation was performed at 300*g* at 4°C for 10 minutes. The resulting supernatant was discarded, and the cell pellet was resuspended in 10 mL of fresh ice-cold complete DMEM, with an aliquot being taken to perform cell counts. Centrifugation was performed again at 300*g* at 4°C for 10 minutes, followed by removal of the supernatant and resuspension of the cell pellet in fresh ice-cold complete DMEM at a concentration of 1 mL per million cells counted. An additional aliquot was taken for cell counts and viability assessments using trypan blue, concomitantly with centrifugation at 300*g* at 4°C for 10 minutes. The resulting supernatant was discarded, and cells were resuspended in ice-cold Cryostor CS10 freezing media (Stemcell Technologies, 07930) at a concentration of 2 million cells/mL. Cells were transferred to cryovials, incubated at 4°C for 10 minutes, and then transferred to an isopropanol cooling container and placed in a –80°C freezer overnight. Cryovials were then transferred the following day to a liquid nitrogen storage tank for long-term storage until all samples were collected. Samples were then rapidly thawed from cryopreservation, washed with 40 mL of ice-cold PBS with 0.5% BSA, and centrifuged at 300*g* at 4°C for 10 minutes. The supernatant was removed, and the cell pellet was resuspended in 200 μL of PBS with 0.5% BSA. Cell counts and viability were determined using trypan blue, and then samples from each treatment group were proportionately pooled to a final count of 5,000 cells for each treatment. Individual samples with less than 80% viability were excluded. This resulted in pooled samples from cell suspensions isolated from 3 animals for each treatment group, which were subsequently used for library preparation.

### Single-cell RNA library preparation and scRNA-Seq.

The isolated mouse pulmonary cells were used for cDNA generation, amplification, and chromium library construction using the 10x Chromium Next GEM Single Cell 3′ Kit v3.1 according to the manufacturer’s protocol (10x Genomics, PN-1000269), which was performed at the Lawson Single Cell Barcoding Facility. Sequencing of the cDNA libraries was performed on the Illumina NextSeq 500 at the London Regional Genomics Centre. The average read depth was 51,767 reads per cell.

### Bioinformatic processing of scRNA-Seq reads.

Sequencer files were converted into FASTQ files and uploaded to the 10x Genomics Cloud Analysis web browser tool. The Cell Ranger Count v7.0.0 pipeline was used to align reads to the reference mouse mm10 2020-A genome and generate feature-barcode matrices for each sample. Output files for each treatment were then combined using the Cell Ranger Aggregate v7.0.0 pipeline. Initial filtering, barcoding, and UMI counting were done using Cell Ranger 7.0.0 and the 10x Genomics recommended default parameters.

### scRNA-Seq data analysis.

Feature-barcode matrices were read into the Seurat package (Version 5.1.0) in RStudio (Version 4.2.2), and analysis was performed. To ensure that cellular barcode data corresponded to viable cells, quality control was performed based on 3 covariates commonly used in the literature (92). First, barcodes were excluded if they contained fewer than 200 genes (likely dying cells) and more than 5,000 genes (likely doublets). Second, cells with greater than 10% mitochondrial transcripts were excluded (likely lysing cells with leaking cytoplasmic RNA). Last, only cells with RNA counts that fell within the ninety-third percentile of all other cells being analyzed were included to omit any major outliers. Data from the remaining cells that passed quality control were subsequently normalized using *LogNormalize* to 1 million reads per cell and scaled. The *FindVariableFeatures* function and variance-stabilizing transformation was used to identify the top 2,000 variable genes. Using Seurat’s *RunPCA()* function, principal component analysis was performed to reduce the dimensionality of the data. Elbow plot and jackstraw plot analysis was used to identify the optimal number of principal components to represent a rigorous compression of the dataset, which was found to be the first 37 independent components. From there, k-nearest neighbor and the Louvain modularity optimization algorithm was used through the Seurat *FindClusters()* function to cluster cells together and generate uniform manifold approximation and projection visualization of the data. Unsupervised clustering was performed using *FindNeighbors* and *FindClusters*, with a cluster resolution = 0.18. The *FindAllMarkers()* function was used with a log fold-change threshold of 0.5 to identify the genes unique to each cluster and establish cluster-specific marker genes. Manual annotation of the clusters was performed using the cluster-specific marker genes in combination with online databases (93–95) and the literature (61, 65). Given their role in the pathophysiological processes known to occur in VILI, namely surfactant dysfunction, inflammation, and microvascular barrier dysfunction, a particular emphasis was placed on analyzing alveolar type II cells, alveolar macrophages, and the capillary cell populations. Differential gene expression analysis between treatment groups was performed using *Seurat’s FindMarkers()* function with either the DESeq2 (96) or the default Wilcoxon rank sum tests. GO pathway analysis was performed using Metascape (97); only genes with an adjusted *P* value less than 0.05, and a log_2_ fold-change greater than 0.5, between groups were included. Last, the CellChat (Version 2.2.2) (98, 99) package in R was utilized to infer cell-cell communication pathways using the provided mouse database. The *identifyOverExpressedGenes* and *identifyOverExpressedInteractions* functions were used to determine overexpressed genes and ligand-receptor interactions. Cell-cell communication probabilities were computed using *computeCommunProb* and *triMean*, with a significance threshold set to 0.05. Unless otherwise stated, all scRNA-Seq analysis performed in RStudio was conducted with the default statistical tests built into Seurat and CellChat.

### Collection of fluids and tissues and biomolecular analyses.

Bronchoalveolar lavage fluid was collected from a separate cohort of ventilated animals and processed, as previously described (34, 100–102). Briefly, lungs were lavaged through the endotracheal tube with 3 boluses of 1 mL of saline. The total lavage fluid was collected and centrifuged at 150*g* for 10 minutes at 4°C to pellet and separate out cells and debris. An aliquot of the supernatant was then collected for protein quantification and assessment of various cytokines by multiplex analysis (MilliporeSigma), including IL-6, CCL2, CSF2, IL-1β*,* CXCL2, CXCL1, IFNγ, IL-10*,* IL-12p70, and TNFα. A separate aliquot of serum from the animals was also collected for analysis of the same cytokines. Lung tissue was collected for qPCR analysis of cytokine and SFTP expression. Lungs from a separate cohort of control animals were also used to measure baseline expression of these cytokines and SFTPs.

### Quantification and biophysical analysis of surfactant.

A separate aliquot of lavage fluid collected above was centrifuged at 40,000*g* for 15 minutes to separate out surfactant subtypes; the supernatant contained the small aggregates while the pellet, which was resuspended in 300 μL of 150 mM NaCl, contained the large aggregates. Quantification of the surfactant phospholipid pool size was performed through lipid extraction using the Bligh & Dyer method, followed by a phosphorus assay, as previously described (103, 104). Large aggregates were resuspended at a phospholipid concentration of 2 mg/mL (140 mM NaCl, 2.5 mM HEPES, and 1.5 mM CaCl_2_ [pH 7.4]), and minimum surface tension was assessed over 20 compression/expansion cycles using a constrained drop surfactometer, as done previously (31, 105, 106). Briefly, 8.5 μL of the surfactant sample was pipetted on the pedestal in an environmentally controlled chamber set at 37°C. A small capillary in the middle of the pedestal was attached to a computer controlled 2.5 mL Hamilton syringe (Hamilton Co.), which was filled with distilled water. After waiting 2 minutes, the surface film of the drop was compressed and expanded through controlled movement of water into and out of the pedestal via the syringe for a total of 20 compression/expansion cycles, at 1 cycle every 1.5 seconds. A camera took pictures of the samples at 15 frames per second as they underwent dynamic changes. Images were then analyzed using the Axisymmetric Drop Shape Analysis computer software program to measure surface tension and surface area based on the shape of the drop throughout the compression/expansion cycles.

### Assessment of pulmonary microvascular permeability in vivo.

Pulmonary microvascular leak was assessed by measuring total protein and levels of the serum exclusive protein, IgM, in the lavage fluid. While lung edema was not directly assessed, both total lavage protein and lavage immunoglobulin levels have been shown to correlate with lung wet/dry ratios, which is commonly used to assess pulmonary edema (62). Total protein in the lavage fluid was determined using a Micro BCA protein assay reagent kit (Pierce Chemical Company, Biolynx Inc.). Total IgM abundance was quantified within the lung lavage fluid by DuoSet ELISA according to the manufacturer’s protocol (Thermo Fisher Scientific, 88-50470).

### PMVEC isolation.

PMVEC were isolated from the lungs of separate cohorts of young and aged nonventilated C57BL/6 mice, as described previously (107, 108). Briefly, the lungs were isolated, finely minced, and digested with collagenase, followed by incubation with CD31 (platelet endothelial cell adhesion molecule [PECAM]) MicroBeads (Miltenyi Biotec, 130-097-418). PMVEC bound to and captured by the microbeads were washed and suspended in DMEM growth medium containing 1 g/L D-glucose, L-glutamine, 110 mg/L sodium pyruvate, and phenol red (Thermo Fisher Scientific, 11885084), supplemented with 20% FBS (Thermo Fisher Scientific, 12483-020) and penicillin-streptomycin (Thermo Fisher Scientific, 15140122). Cells were then seeded in gelatin-coated cell culture flasks and grown to approximately 90% confluence. To assess purity, PMVEC were stained with anti-mouse antibodies against CD31 (BioLegend, 102422), CD34 (BioLegend, 128610), CD146 (BioLegend, 134706), and CD202b (BioLegend, 124010) conjugated to Pacific blue, phycoerythrin, fluorescein isothiocyanate, or allophycocyanin, respectively, and assessed by flow cytometry (easyCyte Guava 12HT; MilliporeSigma). Following this protocol yielded 99% homogeneous PMVEC isolates, which were likely an initial mixture of both capillary type I (aerocyte capillary) and type II (general capillary) cells. Given their abundant proliferation, however, the overall culture probably became enriched for capillary type II cells over time, as these cells are known for their progenitor-like properties (63). PMVEC were cultured for experiments from passages 4 to 10, with media being changed every 2 days.

### Assessment of PMVEC barrier integrity.

Macromolecular flux as a measure of PMVEC barrier integrity was examined, as described previously (107, 109). Briefly, PMVEC were seeded at a concentration of 5.0 × 10^4^ on gelatin-coated 24-well cell culture inserts (3.0 μm pore, VWR) in full DMEM. Cells were grown and monolayer integrity was assessed every second day by measuring transendothelial electrical resistance (TEER; EVOM2 Endothelial Voltohm-meter; World Precision Instruments), with media being changed immediately afterward. PMVEC monolayer TEER was corrected for using an empty insert. The PMVEC barrier was considered to be intact and ready for experiments once the TEER stabilized. Levels of trans-PMVEC macromolecular flux were measured using Evans blue–labeled albumin (BSA, 33.5 μg total in 250 μL; MilliporeSigma), which was added to the upper chamber of the cell culture insert. Following 1 hour, inserts were removed and the conditioned media from the lower chamber were collected and measured through absorbance (620 nm) compared with a standard curve (Victor3 multilabel microplate reader, PerkinElmer Inc.). Values were normalized to average levels seen in the PMVEC monolayers isolated from young animals.

### Immunocytochemistry of junctional proteins.

PMVEC were seeded in 48-well plates coated with gelatin and grown to confluency. Cells were fixed with 200 μL ice-cold methanol for 15 minutes before being washed with PBS. Cells were then permeabilized with 0.1% Triton X-100 (VWR) and blocked using 3% BSA in PBS. The cells were then incubated with antibodies against VE-cadherin (rabbit polyclonal, 1:200 dilution, Abcam, 33168) or claudin-5 (rabbit polyclonal, 1:200 dilution, Abcam, ab15106) in 1% BSA in PBS for 1 hour at room temperature. Cells were washed with PBS and subsequently incubated with Alexa Fluor 594–conjugated IgG secondary antibody (donkey anti-rabbit polyclonal, 1:500 dilution, Invitrogen, A11037) at room temperature for 1 hour. Cells were counterstained using Hoechst 33342 in PBS (1:5,000 dilution; Invitrogen, H3570) and imaged at 200× original magnification by fluorescence microscopy (Zeiss Axiovert 200M Inverted Microscope, Carl Zeiss Canada). Exposure times and contrast levels were kept consistent between the young and aged PMVEC. Five images were taken at random locations within each experimental well, excluding the edges. Image analysis was performed using ImageJ (NIH).

### Statistics.

Data are presented as mean ± SEM. Statistical analyses were carried out using the 2-tailed unpaired Student’s *t* test, 1-way repeated-measures ANOVA, and 2-way ANOVA with Tukey’s test. Results were considered significant at *P* < 0.05.

### Study approval.

All animal experimental protocols, including the implementation of mechanical ventilation, were carried out in accordance with the Canadian Council on Animal Care guidelines for the care and handling of animals and were approved by the Western University Animal Care Committee (Animal Use Protocol nos. 2011-062, 2020-056, and 2020-136).

### Data availability.

The scRNA-Seq datasets generated and/or analyzed during the current study are available in the GEO repository (accession no, GSE274828; https://www.ncbi.nlm.nih.gov/geo/query/acc.cgi?acc=GSE274828). Values for all data points in graphs are reported in the Supporting Data Values file. Other datasets are also available from the corresponding author upon reasonable request.

## Author contributions

AM, LW, CMP, LAM, AT, RAWV, and SEG conducted experiments. AM, LAM, AT, LJJM, SEG, and RAWV analyzed data. AM, AT, RAWV, LJJM, and SEG interpreted results of experiments. CTA, RAWV, and SEG designed research study. AM, AT, OB, RAWV, and SEG prepared figures. AM, RAWV, and SEG drafted the manuscript. AM, AT, RAWV, and SEG edited and revised the manuscript.

## Supplementary Material

Supplemental data

Supporting data values

## Version 1 02/13/2025

In-Press Preview

## Version 2 03/24/2025

Electronic publication
