# Supplementary material for: Effect of aging on pulmonary cellular responses during mechanical ventilation
Source: JCI Insight. 2025 Feb 13;10(6):e185834. doi: 10.1172/jci.insight.185834 (PMC11949020; doi:10.1172/jci.insight.185834)
Supplement: Supplemental data [file jciinsight-10-185834-s211.pdf]

Supplemental Material

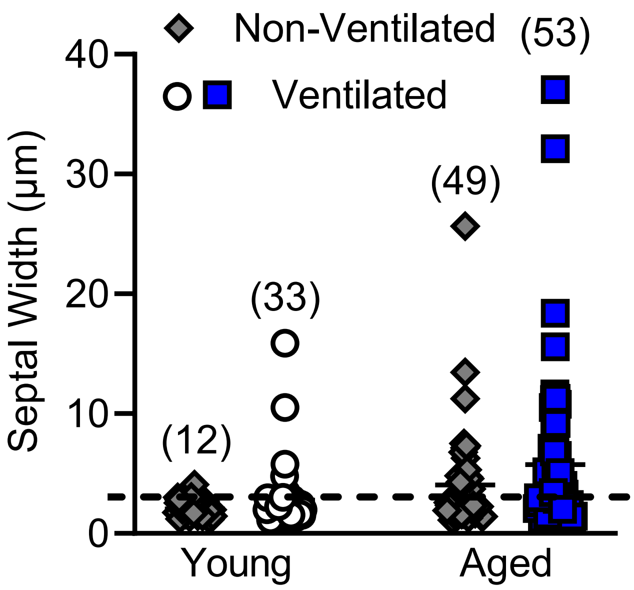

S. Fig 1 Assessment of alveolar septal width in lung histology sections from young and aged non-ventilated and ventilated mice. An increase in septal width and a greater number of thickened septa were observed following ventilation, as well as with age. Dashed line indicates 2.6 μm, the threshold value chosen to delinate a thickened alveolar septum, based on values obtained from the young non-ventilated animals. The numbers in the brackets represent the number of regions that fall over the 2.6 μm threshold value. A total of 12 histology sections were used per animal, with 4-5 animals per group.

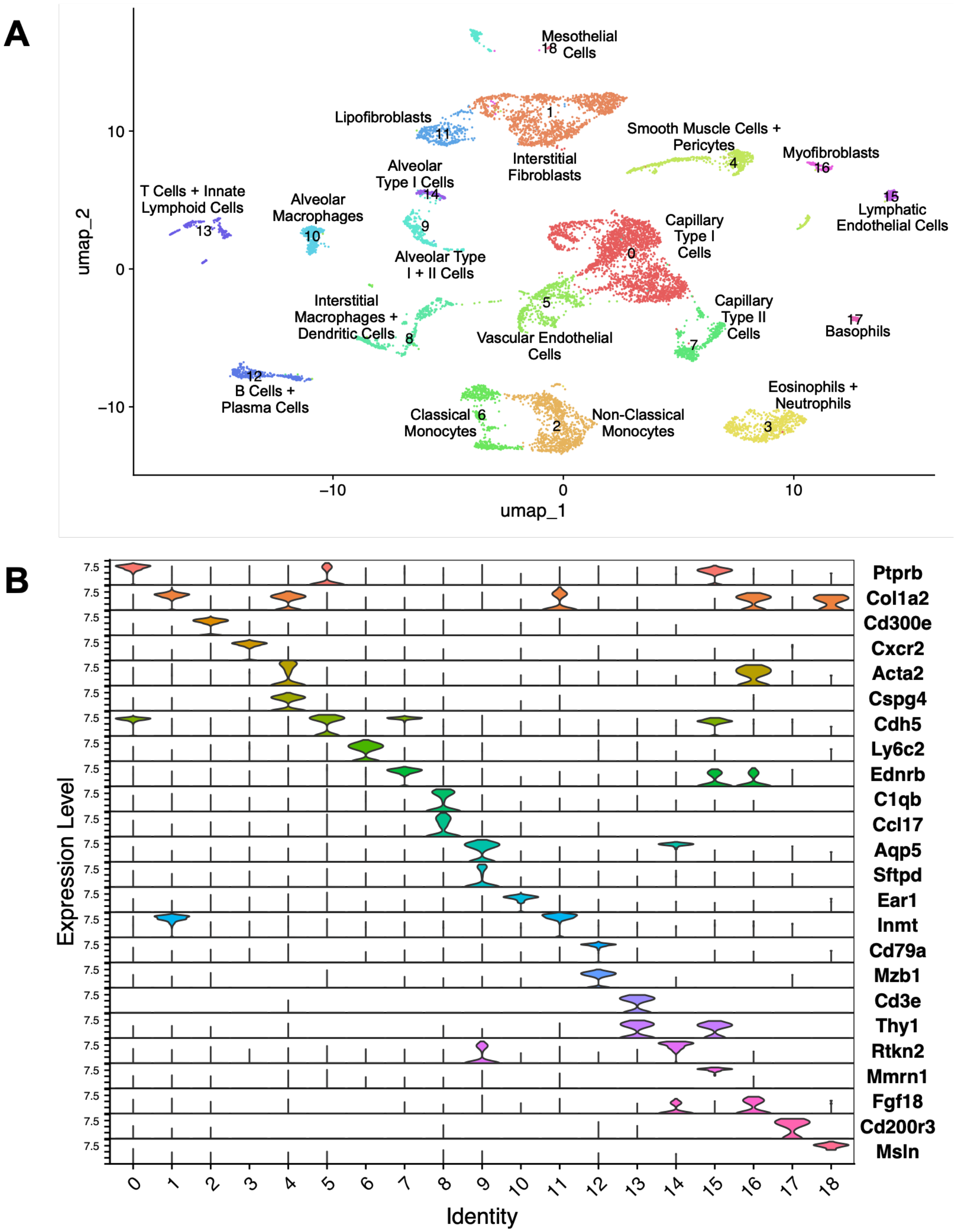

S. Fig 2 Cell identification of single-cell RNA sequencing data of lung tissue from young and aged non-ventilated and ventilated mice. The Uniform Manifold Approximation and Projection (UMAP) visualization of the unsupervised transcriptome clustering; a total of 19 unique clusters were identified (**A**). The stacked violin plot displays the relative expression levels of cells expressing selected marker genes based on unique molecular identifier (UMI) counts (**B**).

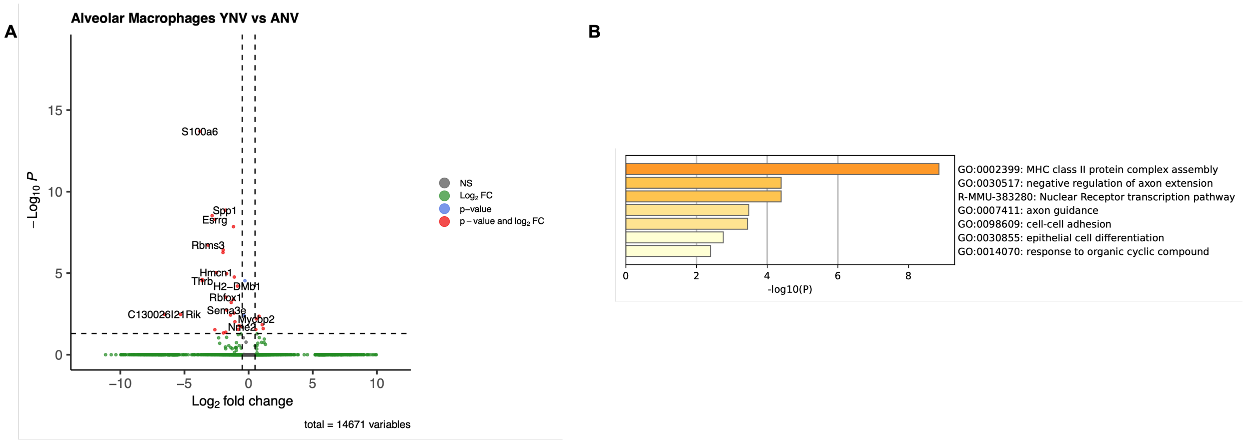

S. Fig 3 Basal differences in expressed genes between the alveolar macrophages from young (YNV) and aged non-ventilated (ANV) mice. The volcano plot reveals 34 significantly differentially expressed genes, most of which are upregulated in the cells from aged non-ventilated animals compared to young non-ventilated animals (**A**). The heat map highlights a number of pathways associated with activation and cell-cell adhesion, based on the differentially expressed genes (**B**). Datasets were derived from n = 3 animals pooled per group.

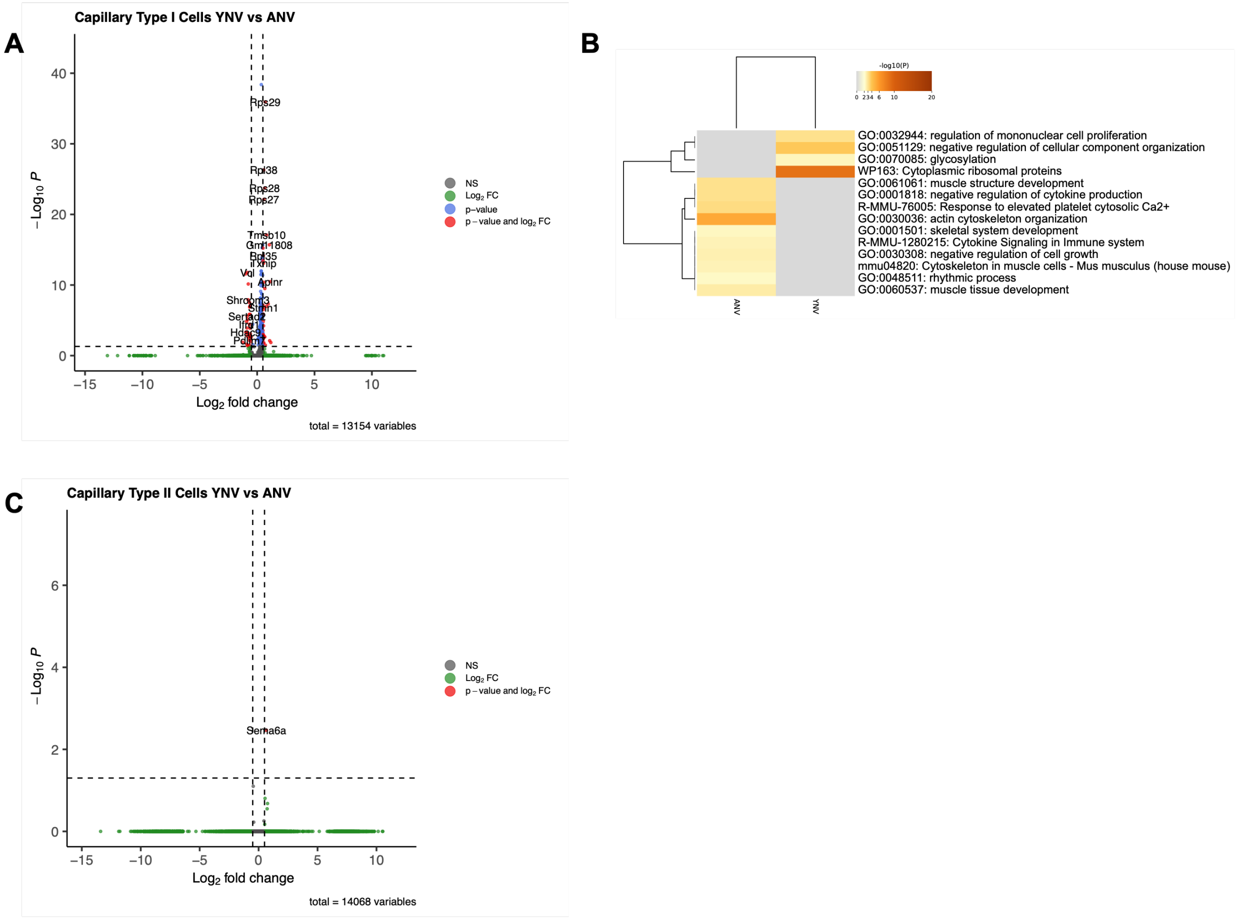

S. Fig 4 Basal differences in expressed genes between the capillary type I and type II cells from young (YNV) and aged non-ventilated (ANV) mice. The volcano plot reveals 64 significantly differentially expressed genes in the capillary type I cells from young non-ventilated animals compared to aged non-ventilated animals (**A**). The heat map highlights a number of pathways describing alterations in signaling associated with the actin cytoskeleton, cytoplasmic ribosomal proteins, and inflammation, based on the differentially expressed genes (**B**). Just one differentially expressed gene was observed in the capillary type II cells between young and aged non-ventilated animals (**C**); heatmap data could not be produced from this. Datasets were derived from n = 3 animals pooled per group.
